# Co‐Design of a Weekly Meal Box for Neurological Conditions: Findings From Consumer and Healthcare Provider Collaborative Workshops

**DOI:** 10.1111/hex.70412

**Published:** 2025-08-28

**Authors:** Joanna Rees, Kelly Moes, Amanda Devine, Melanie Clark, Ros Sambell, Simon Laws, Travis Cruickshank

**Affiliations:** ^1^ Centre for Precision Health Edith Cowan University Perth Australia; ^2^ School of Medical and Health Sciences Edith Cowan University Perth Australia; ^3^ Centre for Culture and Technology Curtin University Perth Australia; ^4^ Nutrition & Health Innovation Research Institute Edith Cowan University Perth Australia; ^5^ Perron Institute for Neurological and Translational Science Perth Australia; ^6^ Collaborative Genomics and Translation Group, School of Medical Sciences Edith Cowan University Perth Australia; ^7^ Curtin Medical School Curtin University Perth Australia

**Keywords:** co‐design, collaborative workshops, meal box, neurological conditions, nutritional requirements, participatory research

## Abstract

**Introduction:**

This study explores the collaborative co‐design process for developing a weekly meal box tailored for individuals with neurological conditions. Recognising the critical role of nutrition for this community, the research addresses the challenges posed by cognitive and physical impairments in meal preparation.

**Methods:**

Through two co‐design workshops involving consumers, healthcare providers and industry experts, insights were gathered on dietary preferences, nutritional needs and practical challenges.

**Results:**

The workshops emphasised the importance of convenient, easy‐to‐prepare meals with simple instructions, flexibility and customisability. Consumers expressed preferences for convenient, easy‐to‐prepare meals with simple, easy‐to‐follow recipe instructions that align with optimal dietary patterns and taste preferences. Health and industry experts emphasised the importance of flexibility/customisability, ease of access and meal preparation. A prototype meal box was developed and tested in a simulation event, revealing positive feedback and areas for improvement. Participants appreciated the pre‐prepared ingredients and reported increased confidence in cooking.

**Conclusion:**

By involving both consumers and health and industry experts in the design process, this study contributes to the design of meal box solutions that have real potential to improve the quality of life for those managing neurological conditions through nutrition. The co‐design approach ensured the meal box met the specific needs of the target group, promoting sustainability and practical application. Future research will focus on refining the prototype and evaluating its effectiveness in a broader pilot study. This study underscores the importance of user‐centred design in creating viable nutritional solutions for individuals with neurological impairments.

**Patient and Public Contribution:**

People with lived experience of a neurological condition, their carers, health providers and industry experts contributed throughout the design process and the preliminary simulation event. Our thematic analysis was conducted by someone with lived experience of a neurological condition, who also contributed to the writing and reviewing of the manuscript.

## 1 Background

Neurological disorders are disorders of the brain and nervous system, such as epilepsy, multiple sclerosis (MS), Parkinson's disease and acquired brain injury (ABI) [1]. Maintaining a healthy diet is recommended as a priority for more than one in three people globally who are living with neurological conditions (National Institute on Ageing [2, 3]). However, a large proportion of this population has cognitive and motor impairments (e.g., decreased hand dexterity), which compromise their ability to procure, prepare and cook healthily [4]. Complications getting to and from the shops to purchase ingredients and equipment for cooking, as well as the development of swallowing difficulties, further compromise their nutritional intake.

The provision of weekly meal boxes and recipe kits in the community is becoming increasingly popular and represents a viable solution to access good‐quality food [5]. Such meal boxes have the potential to reduce the burden associated with meal procurement and preparation and improve nutritional intake. However, the content of meal boxes that are currently available varies greatly in the context of nutrition quality, recipe complexity and cost value [5]. Furthermore, most meal boxes include ingredients that require preparation, such as peeling and chopping of whole fruits and vegetables. Regardless, this practice has the potential to improve nutritional intakes within the community, particularly if meal boxes were to replace less nutritious convenience‐based ready‐to‐eat meals [5].

Research shows that diets like the Mediterranean and MIND (Mediterranean‐DASH Intervention for Neurodegenerative Delay) are beneficial for people with neurological conditions, helping delay progression and improve quality of life [6, 7, 8]. These diets incorporate leafy greens, berries, nuts, whole grains, fish and olive oil, with minimal amounts of ultra‐processed, refined foods, red and processed meat, and unhealthy fats [9, 10, 11]. The diets provide rich sources of plant‐derived antioxidants that focus specifically on brain health, as well as being high in dietary fibre to support the gut microbiome and the gut–brain axis [12, 13]. A tailored weekly meal box offers a vehicle by which people who are living with a neurological condition may access the benefits of a Mediterranean/MIND diet.

Previous nutrition interventions trialled in the research setting have reported underlying issues such as poor long‐term adherence and a lack of translation into the commercial setting [8, 14, 15]. To overcome this, fostering integrated knowledge translation and a collaborative approach could present a feasible solution. Moreover, engaging those with lived experience to identify individual experiences, needs, priorities and values to inform the development and testing of an intervention in the health care setting has proven to be effective [16, 17].

To our knowledge, no published studies have examined the use of weekly meal box provision comprising healthy fresh ingredients that are pre‐prepared and appropriate for people with neurological conditions, who experience impaired motor and cognitive function, fatigue, apathy and gastrointestinal issues. The provision of meal boxes with pre‐prepared ingredients offers the opportunity to include supplementary ingredients with additional nutrients if required, as well as additional resources on healthy eating to improve food literacy. Additionally, it can be tailored to meet the specific needs of a particular neurological condition, as these may vary. This concept greatly reduces the need to navigate busy grocery stores and the mental burden associated with meal planning and selecting nourishing recipes and appropriate ingredients. Finally, when compared with grocery‐procured meals, meal boxes have been found to address sustainability goals by creating less food waste at the consumer level [18] and producing lower carbon [19] and greenhouse gas emissions [20]. Pre‐prepared meal boxes offer further sustainability by presenting an opportunity to collaborate with local producers and to valorise ‘ugly’ or damaged produce that would otherwise be wasted [21].

### 1.1 Aim

The overarching aim of this project was to employ a collaborative approach to co‐design and test a weekly meal box, specifically tailored to the needs of people living with a neurological condition. The objective is to capture the needs and preferences of consumers, health professionals and industry partners (IPs) specific to the neurological community. We hypothesised that this service could help overcome the burdens experienced surrounding meal procurement and preparation, be inclusive and meet the disability and dietary needs of people living with a neurological condition. In turn, this would help to maintain activities of daily living, whilst improving nutritional intake and quality of life. The current project forms part of a larger programme of work to develop, validate and translate this study into a service that is readily available to the neurological community.

## 2 Methods

### 2.1 Project Design

**Figure 1:**
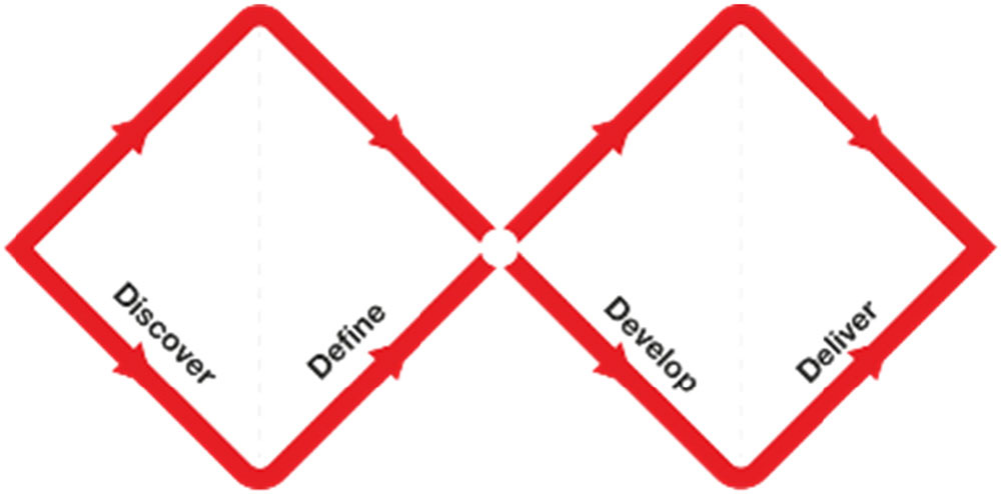
The double diamond [23].

The initial steps of the project follow IAP2 core values [22] and have employed participatory research techniques that involve end users and professional stakeholders (people living with a neurological condition and caregivers, as well as researchers and allied health professionals) to be involved from the outset to better understand the perspectives of those who will use and recommend the meal box service. The project follows the British Design Council's Double Diamond Design Process (DDDP) [23] in which there are four overlapping stages (Figure 1).

1. **Discover:** this involved three steps.

a. To inform the work and build on from existing learnings, a literature review was conducted to find out whether there was any existing research for the provision of a weekly meal box tailored to the neurological community (Supporting file 1) and none was found.
b. Find out what the consumers' and health and industry experts' (HIE) broad hopes and fears surrounding a potential weekly meal box service would look like and determine what motivators and barriers would need to be addressed.
c. Learn the perceptions of existing meal box providers and find out if there was a potential to recruit an interested IP.

2. **Define**: Narrow down to the most important issues that need to be considered, beyond the ingredients and recipes that need to be included, such as delivery, packaging, ordering and receival and possible inclusion of additional items such as assistive devices.
3. **Develop**: Work with an IP to develop the prototype recipes, assess how they would need to be modified and ingredients pre‐prepared and whether this would be economically viable, practical and achievable.
4. **Deliver**: Introduce the initial prototype design with a group of community members to inform on any refinements that would be necessary before conducting a pilot feasibility study with a small group of participants from the neurological community.

The formative **phase 1** of the project brought together consumers, health and industry professionals, and key stakeholders who applied their lived experiences and expertise to the co‐design of a weekly meal box. Co‐design, where those with lived experience and relevant stakeholders are involved in the research process, has been shown to be effective in public healthcare outcomes and lead to more sustainable and acceptable local solutions to local problems [24]. **Phase 2** of the project utilised the data captured from the workshops, which informed the development of an appropriate and desirable prototype meal box tailored to the needs of those living with a neurological condition. This prototype was tested in a simulation event.

Consumer involvement was positioned at the ‘collaborate’ level of the IAP2 Public Participation Spectrum, with people with neurological conditions and their carers participating as partners in workshops during the **discover** phase, whilst an individual with neurological conditions led the data analysis and follow‐up interviews in the **define** phase. This approach ensured shared decision‐making and balanced power dynamics throughout the co‐design process, with consumers actively shaping both data collection and interpretation.

### 2.2 Phase 1—Co‐Design Workshops

For the co‐design process, two workshops were held: a consumer workshop which comprised people living with a neurological condition and caregivers; and a workshop which comprised HIE, including meal box providers, healthcare professionals (dietitians, speech pathologists, occupational therapists, psychologists and physiotherapists) and researchers specialising in neurological conditions and nutrition. These workshops formed the **Discover** stage of the DDDP, their purpose being to introduce the concept to the overall community and then promote and capture individuals' ideas and perceptions to create their ideal visual prototype of the meal box.

#### 2.2.1 Participants

Consumer workshop participants were recruited via existing research connections, networks and databases, as well as relevant not‐for‐profit organisations. This group are referred to as consumers. Inclusion criteria specified people over 18 years old who were living with a neurological condition or supporting someone with a neurological condition. Participants for the HIE' workshop were recruited via existing research, health service and industry expert networks, existing meal box providers and local producers. This group are referred to as HIE. For both workshops, potential participants were sent a flyer with workshop details and a link to register. Before attending the workshops, all interested individuals were sent a detailed information letter and provided written consent. Talent release forms were signed to provide permission for all photographs displayed. The study has been granted ethics approval.

#### 2.2.2 Workshop Format

The workshops were designed by the research team (RT) and piloted by members of the community with lived experience and professional stakeholders before delivery. These pilot sessions helped inform and confirm the approach. People with lived experience indicated that in‐person workshops were better in pilot sessions. Professional stakeholders indicated that Miro was a suitable platform to run workshops. Both groups provided feedback on the time allocated to each activity and how to structure the workshop session, with regular breaks, food and drinks, and additional support provided for people living with a neurological condition, given fatigue, physical, cognitive and behavioural symptoms, also to facilitate a safe environment for the workshop. Professional stakeholders indicated running the workshop at a time conducive to national and international stakeholder engagement and keeping the workshop time short to encourage participation.

Workshops were guided by human‐centred design (HCD) principles [25], with the intervention co‐designed alongside people living with neurological conditions and their caregivers. The process was also informed by the insights of professional stakeholders, including researchers, dietitians, occupational therapists, speech and language therapists, public nutritionists and neuroscientists, ensuring the approach was clinically relevant and context sensitive. While the workshops were not theory‐driven, relevant theoretical models such as the COM‐B model (Capability, Opportunity, Motivation Behaviour) were considered during data analysis to help interpret the behavioural, cognitive and physical factors influencing participants' engagement with cooking and nutrition. We acknowledge concerns that theory‐led design can constrain creativity, bias interpretation or limit attention to insights that fall outside predefined frameworks. For this reason, theory was used reflectively to support the interpretation of participant insights rather than to drive the design process, preserving the core values of inclusivity, empathy and co‐creation central to HCD [25].

Consumer workshops were undertaken in person, while HIE workshops were carried out online, with national and international experts joining. An in‐person format was deemed the most practical for consumers, to avoid excluding those who may not have access to, or the skills necessary for, an online format. In contrast, to enable national and international participation and capture a broader perspective, the HIE workshops were conducted online. Both workshops lasted about 1–1.5 h and were facilitated by J.R. and T.C. with support from M.C. and A.D. Additional research staff and students assisted with scribing, where needed (particularly for the consumer workshop).

**Figure 2:**
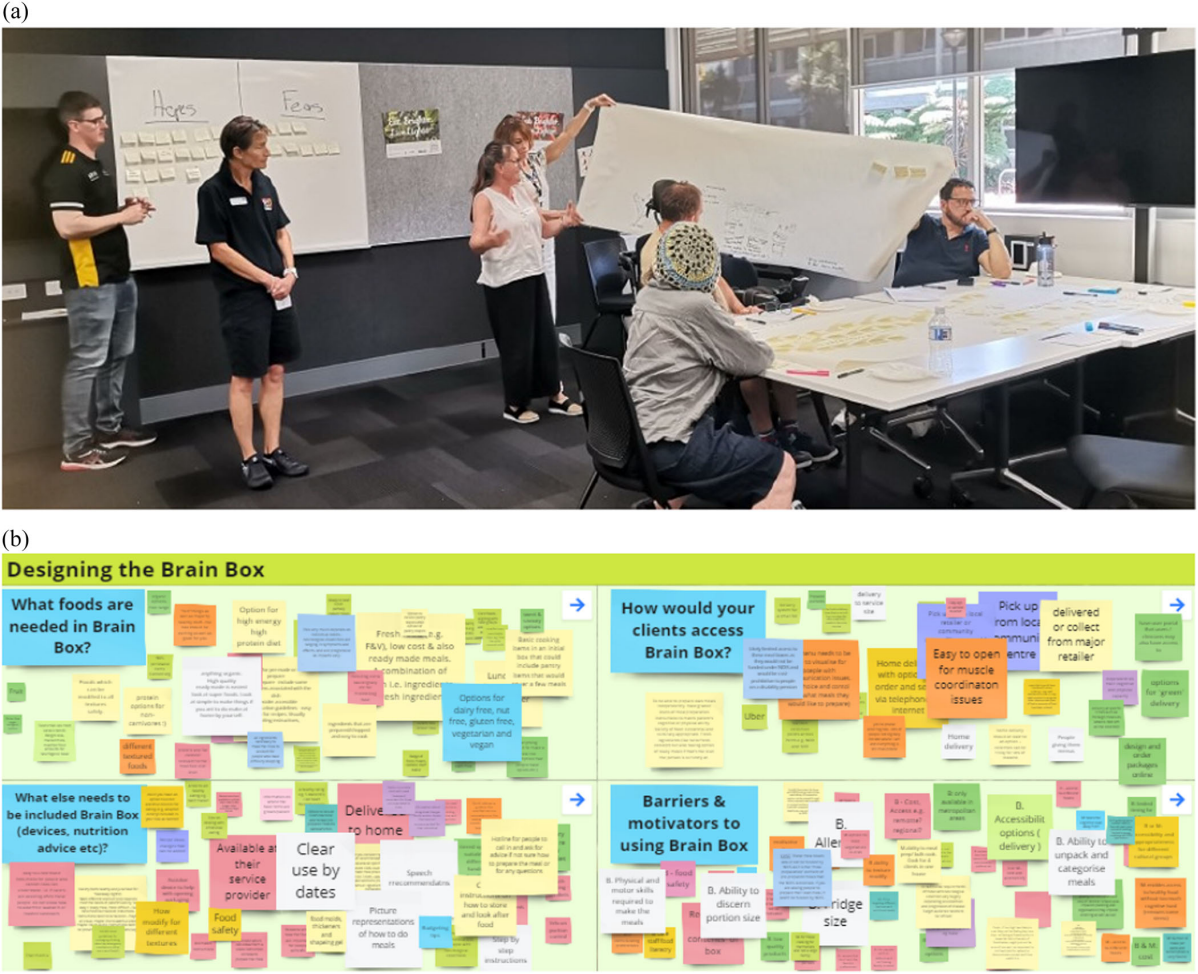
(a) Consumer workshop. (b) Industry expert workshop Miro Board [26].

The consumer workshop was conducted in an all‐access room suited to the purpose at the RT's location in Perth, Western Australia. Participants shared their views and perspectives via physical contributions through sketches and comments on sticky notes, butcher's paper and whiteboards (Figure 2a). All content, thoughts and ideas for simple prototypes throughout workshops were extracted into Excel spreadsheets and Word documents for content analysis.

The HIE workshop followed the same format as the consumer workshop, but via an online modality, Miro [26] (an online whiteboarding software). The views and perspectives of participants were captured via virtual contributions on sticky notes, comments and pictures, captured in online wireframe sketches (Figure 2b). The workshop was facilitated by the same four members of the RT who were involved in the consumer workshop.

For both workshops, the first two activities were conducted as a whole group, after which participants were divided into four breakout groups for the third activity, before coming back together as a whole group for the final activity, see schedule below. Each breakout group was supervised by a member of the RT. Topics for discussion followed the sequence described below.

▪ Activity 1. (Whole group) Hopes and fears regarding nutrition, mealtimes, eating habits and practices.
▪ Activity 2. What foods should be included? → How should the box be accessed? → What else should go in the box? → What barriers and motivators are there to encourage or deter a meal box service?
▪ Activity 3. (In four breakout groups) Create a prototype meal box using the feedback given so far.
▪ Activity 4. Present your prototype to the rest of the workshop participants.

All information captured on the sticky notes and wireframes was collated using the Miro software program into Excel spreadsheets for analysis.

#### 2.2.3 Data Analysis

Qualitative content analysis was employed during the ‘discover’ and ‘design’ phases of the co‐design process to systematically examine participant responses [27, 28], focusing on manifest content to identify key features and patterns related to meal box development. The analysis proceeded through three iterative stages. Initial coding involved generating codes based on predetermined categories derived from workshop questions, with additional emergent codes added to capture unexpected participant expressions. These codes were then developed into broader thematic categories through a process of grouping and organising related concepts, utilising subcategories as intermediaries to facilitate this organisation. The researcher moved in a non‐linear way through the data, identifying connections and patterns whilst considering both participant perspectives and contextual factors. Codes were reviewed to ensure the themes adequately captured the data and refined into categories to clarify what each group represented [29]. Finally, thematic frequency was analysed to prioritise the most pressing user concerns, aligning with the translational and intervention‐focused objectives of the co‐design approach. This method enabled a descriptive analysis that allowed both explicit and implicit perspectives across our diverse participant groups to be captured without seeking deeper interpretive meanings, thereby maintaining focus on practical insights for meal box intervention development.

### 2.3 Phase 2—Prototype Development and Test Run

#### 2.3.1 Prototype Development

The visual prototypes from phase 1 were refined and transformed into a viable prototype for testing in a simulation event to establish initial feasibility before a pilot trial (**Define** stage of the DDDP). In alignment with the **Define** and **Develop** stages of the DDDP, the RT worked together with their IP, an existing meal box service provider (identified via the HIE workshop), to synthesise and refine the visual prototypes from both the consumer and HIE workshops. In consideration of the concepts outlined in Table 1, a physical version of the meal box was created. The IP's recipe database was examined, and those which most closely adhered to Australian Dietary Guidelines (ADG) [30] and included ingredients recommended by the Mediterranean or MIND diets were selected.

**Table 1:** Prototype development schedule undertaken by the research team (RT) and industry partner (IP)'s business manager and chef.

Element	Tasks	Action
Recipes	Choose the recipes for the 10 meals (2 × 5 main meals) ADG/MIND/Mediterranean ≤ 30 min to complete, involve little equipment, simple and easy	RT
Pre‐prepared ingredients	Suggest modifications	IP
	Determine feasibility, sourcing, time economy, extra costs incurred, logistics	IP
Ingredients	Shelf life Appropriateness Quality/quantity Packaging (sustainable) and easy to open	IP
Recipe instructions and options	Pictures	IP
	Choice/selection—protein choice, etc. Adaptability	IP and RT
Temperature control	Ice packs Insulating sleeve	IP
The box	Order/delivery process Weight of box How packed	IP and RT
Inclusions—Assistive devices/information	Tools Nutrition information, education	RT

Abbreviations: ADG, Australian Dietary Guidelines; IP, existing meal box provider industry partner; MIND, Mediterranean‐DASH Intervention for Neurodegenerative Delay; RT, research team.

#### 2.3.2 Test‐Run Participants

Aligning with the **Deliver** stage of the DDDP, a simulation event was conducted to test the feasibility and gather feedback on the meal box from the perspective of the consumers and the facilitators, that is, the RT and the IP who provided the prototype meal boxes. This event was attended by two individuals living with ABI, accompanied by their support person or the person they most usually cooked with, as well as the IP's business manager and head chef. The ABI participants were identified through the RT's networks.

#### 2.3.3 Simulation Event Format

Participants and their support person were invited to attend the RT's cooking facility to test run the developed prototype. The event involved providing feedback on receiving, unpacking, preparing, cooking and eating one of the specially modified recipes. The support person (spouse/support worker) assisted the participant, when needed, with cooking activities. Each participant was asked to complete a checklist throughout the session to capture their experiences with the various stages of the meal box. These included receiving and unpacking a meal box; identifying the ingredients for their recipe; and following the recipe instructions to prepare and cook the recipe, which provided ingredients for two people. Finally, participants and their support person shared the meal they had cooked and provided their feedback on the taste of the meal and the overall experience. Details of the checklist are displayed in Supporting File 2. The IP's business manager and head chef were present to experience feedback from the consumers first hand and respond to any questions that arose throughout the process.

## 3 Results

### 3.1 Phase 1—Co‐Design Workshop Results

Workshop participant characteristics are displayed in Table 2. Demographic details such as age, sex and ethnicity were not collected; however, participants in the consumer workshop included those from varying ethnic backgrounds but were all residing in Perth Western Australia. The online format of the HIE enabled the participation of health professionals from interstate Australia and the United Kingdom, as well as Western Australia. The HIE workshop enabled the RT to identify an interested IP with whom they established a working partnership.

**Table 2:** Workshop participants.

Consumer workshop	Participants (*n*)	Health and industry expert workshop	Participants (*n*)
Acquired brain injury (ABI)	7	Speech pathologist	4
Multiple sclerosis (MS)	4	Dietitian	4
Encephalomyelitis	1	Occupational therapist	2
Intracranial hypertension	1	Psychologist	1
Functional neurological disorder (FND)	1	Social worker	1
Carer (MS)	1	Care services coordinator	1
		Researcher	1
		Meal box service provider	2
		Producer	1
Total	15		17

#### 3.1.1 Code Development

**Figure 3:**
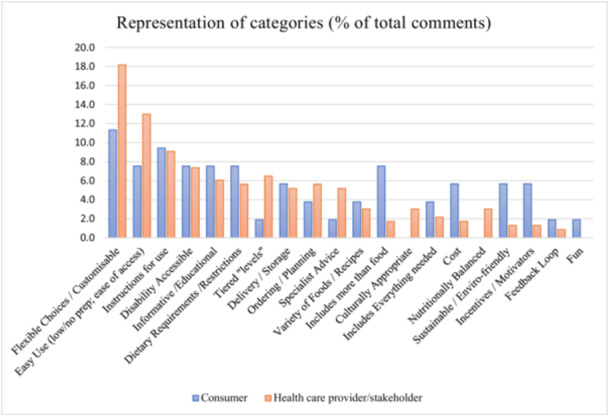
Representation of categories for the development of the prototype meal box from both workshops as percentages of total comments for each workshop.

After initial inspection of the data, codes for hopes and motivators were combined and labelled ‘Positives’, while codes for fears and barriers were grouped and labelled as ‘Negatives’. Categories were then determined for these and the remaining topics comprising Activity 2, by categorising/grouping key words and noting patterns across the data. Finally, the most common categories were identified for the suggested prototype meal boxes (Figure 3). The development of codes and categories was informed by both predetermined frameworks and emergent participant expressions. For instance, the ‘culturally appropriate’ category emerged from multiple participant references to cultural and religious dietary requirements that extended beyond basic food allergies or preferences. Participants specifically highlighted concerns about meal boxes ‘not being inclusive for dietary needs/allergies/cultural requirements, for example, halal’, meals being ‘designed for a local majority and not appropriate for minority groups’, and services ‘not understanding community’ needs. These expressions were grouped together as they shared a common focus on cultural inclusivity and community‐specific requirements, rather than individual dietary preferences. This category was distinguished from the broader ‘dietary requirements’ code by its emphasis on cultural identity and community belonging, reflecting participants' need for meal services that recognised and respected diverse cultural practices and food traditions within the neurological disability community. This approach demonstrates how codes evolved from surface‐level content analysis whilst maintaining sensitivity to the deeper cultural and social contexts that participants identified as important for meal box accessibility and acceptability.

#### 3.1.2 Activity 1—Hopes and Motivators (Positives); Fears and Barriers (Negatives)

The workshop participants were asked to describe what their ideal meal box should or should not include, imagine how it would look in a perfect world, and consider the factors they envisaged would prevent this or need to be overcome. Overall, the most important responses identified by both the consumers and the HIE were about usability, that is, that the meal box be practical and simple and provide quality, nutritious food.

Positive example quotes from consumers (C) included ‘*easy meals’* where you ‘*don't have to think about what to eat and how to make it’, ‘easy recipes with low prep’* and ‘*easy‐to‐follow steps’*. The HIE also emphasised the process needed to consider the cognitive and physical capabilities of consumers but be ‘*visually appealing enjoyable food choices nutritionally complete easy to cook’*. This ‘*enables access to healthy food without too much cognitive load (removes some stress)’* and builds the ‘*ability to create easy to make food/meal* (HIE)’ that are modified for neuro‐specific populations.

Negative issues raised by consumers included the need for the food to ‘…*suit the whole family’* and not be ‘*only personalised for one…’*, whereas HIE were concerned with practical issues such as ‘*complex recipes’* and ‘*food packaging is too hard to open’*.

#### 3.1.3 Activity 2—What Foods Should Go in the Box?

Results from the consumer workshop showed that the inclusion of fruit and vegetables, ‘*fresh locally produced vegetables and fruit’*, was the most important food that should be included in the box, followed by consideration of specific dietary needs/preferences ‘*nut‐free/dairy‐free/gluten‐free/vegetarian options’* (HIE). The latter was the most highly represented topic from the HIE workshop.

– How should the box be accessed?
  Both workshops considered home delivery to be the most suitable method of accessing the meal box with a pre‐organised set delivery time ‘*delivery when I'm home’* (C) and a simple‐to‐use ordering system, ‘*easy app or website to order’* (HIE).
– What else should go in the box?

Easy‐to‐follow visual/pictorial instructions ‘*diagrams showing how to construct meals on the plate’ (C) ‘visual (pictorial) menu options’* (HIE) were the most important features for both consumers and providers, followed by the ability to modify the recipes and ingredients to suit different needs ‘*pre‐prepared ingredients’* (C), *‘menu choices with different levels of prep options to meet patient's ability’* (HIE). Other topics that were deemed important included simple and easy‐to‐follow instructions and how to use the meal box, that is, ‘*clear instructions on how to store and look after food’* (HIE), the inclusion of nutritional information ‘*how nutrients affect my body’* (C), ‘*how the food helps your brain’* (C), and if needed, information regarding texture modification of foods including ‘*clear dietary guidelines re textures/consistencies’* (HIE).

#### 3.1.4 Activities 3 and 4—Prototype

**Figure 4:**
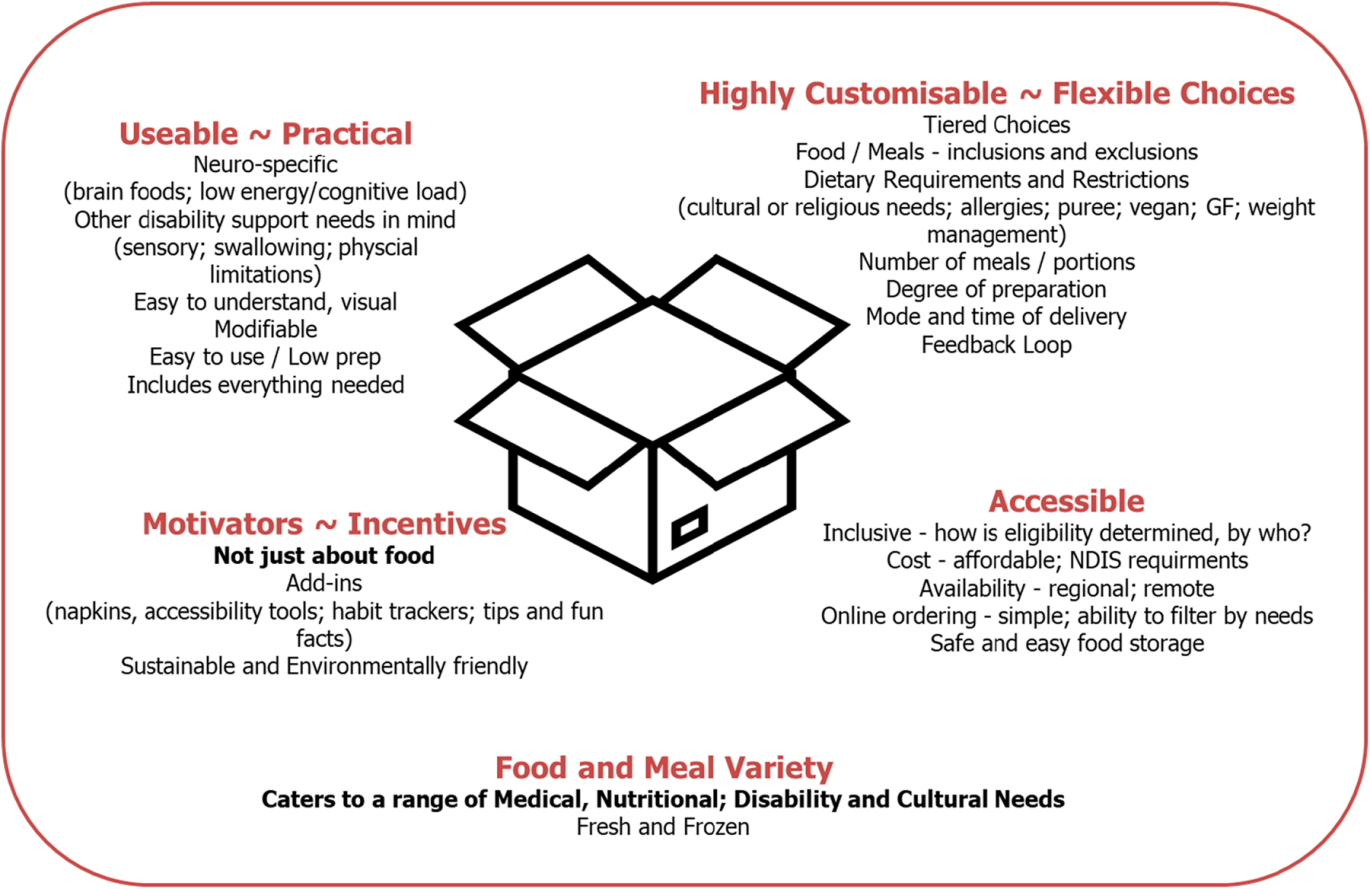
Summary of key thematic codes (in red) and subcategories (in black) arising from the two workshops.

A summary of the overall findings arising from both workshops is displayed in Figure 4. The key findings (in red) were used to guide the development and delivery of the prototype meal box.

### 3.2 Phase 2—Test‐Run and Simulation Event Results

The simulation event was attended by two volunteer participants with an ABI accompanied by their support person who were identified via the RT's networks. Facilitators included the IP's business manager and head chef and five members of the RT. Overall, five different meals were prepared, cooked, eaten and reviewed with feedback captured throughout the process. Feedback from the simulation event was overwhelmingly positive and highlighted the feasibility and potential benefits of the meal box prototype while also identifying areas for further improvement.

Participants showed enthusiasm and engagement throughout the event and showed their appreciation for the thoughtfulness that had gone into the meal box design, especially features like pre‐chopped ingredients. Both participants reported that the experience increased their confidence to cook. They were grateful that someone was listening to their daily challenges and excited about the possibility of meal boxes as a practical solution in the future.

A summary of the key observations deduced from participant and facilitator feedback, both conversationally and via feedback questionnaires, was as follows (full details provided in Supporting File 3):

– The event created a supportive, welcoming and enjoyable atmosphere.
– Participants actively provided feedback and suggestions for improvement.
– The cooking experience was empowering, especially for those who had previously given up cooking due to their cognitive and physical impairments.
– The event helped facilitators understand challenges like being ‘nonverbal’ post‐accident and the resilience of individuals overcoming trauma.

A summary of the positive outcomes and challenges identified from the simulation event is provided in Table 3.

**Table 3:** Key outcomes from the simulation event.

Challenges/Concerns identified	Positive outcomes
Initial anxiety about cooking independently for some participants.	Demonstrated that participants could cook meals independently.
The cost of meal boxes could be a barrier to adoption.	Highlighted the potential benefits of hosting an induction/simulation event to help overcome any issues before a future feasibility intervention study.
People with more severe disabilities might struggle to use the meal box.	Provided valuable feedback for further improvements to the meal box (e.g., dividing ingredients into two boxes, making packages easier to open and including more steps in the instructions).
Weight of the box (suggestion to split food into two boxes).	Created a good ratio for stimulating conversation and allowing researchers to listen to participants' stories.
Executive function issues (suggestion to provide guidance on meal planning).	Strengthening the co‐design feature of the meal box creation.
Identified that some packages were hard to open	

## 4 Discussion

To our knowledge, this is the first study to co‐design a meal box solution for the neurological community or any clinical community. The benefits of co‐design have been well documented, particularly for the design of sustainable products or solutions that can effectively be translated into everyday practice [24]. This project combined the hands‐on experience of those living with a neurological condition with the expert opinions of HIE and a highly experienced RT. Findings from the two collaborative workshops provided invaluable insight into how a weekly meal box that is tailored to the neurological community should look and what it should contain. Most importantly, the concept was positively received by both those living with a neurological condition and the professional community who provide their healthcare. The themes identified in the workshops were integral in the following steps of the project and were factored into the prototype design. Furthermore, and of critical importance, the HIE workshop enabled us to connect with an established meal box provider who was willing to collaborate with the RT on this project. This was fundamental, as engagement with an industry provider is essential for translating the concept into a community setting, ensuring its successful implementation and long‐term sustainability.

Although still relatively under‐researched, meal provision through home‐delivered meal boxes is an emerging trend that is gaining attention in research and practice. There is mounting evidence that the service encourages family connectedness and supports engagement in food‐related tasks in general [31]. Furthermore, a weekly meal box service helps to reduce the burden surrounding menu planning and the need to navigate grocery stores to acquire nutritious ingredients [32]. This is of particular benefit to those in the neurological community for whom these activities raise extra logistical challenges [15]. There are limited nutrition interventions available for people living with neurological conditions, and those that exist are frequently not sustainable [14, 15]. Typically, the nutritional advice provided relies on the individual or their family member following guideline documents for certain diets, having to research suitable recipes to follow and then acquiring and preparing ingredients [15]. This often presents too great a cognitive and/or physical burden such that unhealthy options are frequently chosen as an easier alternative, including the purchase of ready‐to‐eat processed foods [31, 33]. Individuals who engaged in the research process warmly received the concept of being able to circumnavigate the challenges of trying to follow healthy eating guidelines. The provision of nutritious recipes that adhere to the ADG [30] with easy‐to‐follow, clear, concise pictorial instructions, as well as pre‐prepared fresh ingredients and less than 30‐min cooking times, were some of the most important points raised in the workshops. The simulation event demonstrated that these modifications were well‐received and contributed greatly to the success of the prototype meal box.

### 4.1 Benefits of This Study

Currently, evidence‐based solutions to support individuals with neurological conditions in maintaining long‐term engagement in the kitchen and sustaining healthy dietary habits are scarce. Yet being able to perform activities of daily living and ongoing self‐management has been proven to provide meaning and purpose in life that is central to an individual's psychosocial well‐being [15, 34]. Taking this into account and in collaboration with individuals with lived experience, subject matter experts and IPs, this study has co‐designed a meal box tailored to the dietary and accessibility needs of people living with neurological conditions. This solution leverages the rapidly growing meal box industry and commercially available product streams, ensuring translation for the community.

This study reinforces the importance of user‐centred design and community engagement. It has provided initial validation for the meal box concept, plus the associated potential benefits of improved nutrition and quality of life for people with neurological conditions. The consumer workshop highlighted the need to consider issues such as meal box delivery, how the box will get from the doorstep into the kitchen and that for some, unpacking the box may be difficult. This was further demonstrated at the simulation event when a single box was too heavy, and meals would be delivered in two boxes in the future. Valuable insight captured from both the workshops and the simulation event identified that special consideration must be given to making the packaging easy to open whilst still maintaining the ingredients' safety and freshness. Continued consumer involvement ensures that the resulting meal box service meets the needs of the neurological community, which is critical if the service is to become a sustainable resource in the future.

Whilst this study targets the broader neurological community, there is much potential for distinct iterations of the meal box to be tailored to specific conditions such as Huntington's Disease, as just one example. The findings could provide case study insights for advocacy to garner support for modified meal boxes and beyond.

### 4.2 Limitations

We had unequal representation of neurological conditions and health providers in both workshops, and only specific neurological populations were represented. In addition, the inclusion of individuals from different cultural backgrounds was limited, and all participants in this study spoke English. Therefore, the generalisability of findings may be limited. Only two people with an ABI and their support people were invited to participate in the initial prototype evaluation, limiting the diversity of perspectives and the applicability of findings across broader neurological populations. While a meal box service won't address every challenge, it has the potential to ease cognitive and physical burdens around daily meal preparation.

### 4.3 Future

The findings presented in this study are the result of a 2‐year collaboration with key stakeholders, including individuals with lived experience, health professionals, researchers and an IP. Participants in the simulation event reported a rewarding experience, as it reinforced their confidence in their culinary abilities and their capacity to engage in meal preparation. Insights from this study have informed subsequent phases of the project, facilitating ongoing collaboration between the RT, the IP and the neurological community to refine the meal box prototype. This iterative process aims to incorporate the critical features identified while also ensuring the solution remains sustainable and economically viable. The meal box has been trialled in a small pilot study with volunteer families, each with a member living with a neurological condition. Findings from this pilot study will inform further refinements before progressing to a randomised controlled trial to assess feasibility and gather preliminary efficacy data.

## 5 Conclusion

By involving both consumers and HIE in the design process, this study sets the foundations for enhancing the quality of life for those managing neurological conditions through nutrition. Findings will enable rapid implementation within the neurological community through an existing meal box provision service in Perth, Western Australia. Future optimisation of the meal box, involving continued participatory research, will guide progression. Ultimately, the goal is to make continuous improvements and develop a customisable meal box service that supports in‐home meal preparation, empowering individuals with differing neurological conditions to maintain independence and meet their dietary needs.

## Author Contributions

**Joanna Rees:** conceptualisation, writing – original draft, review and editing, project administration. **Kelly Moes:** participation, thematic analysis, writing – original draft, review and editing, **Amanda Devine:** conceptualisation, writing – review and editing. **Ros Sambell:** writing – review and editing. **Melanie Clark:** participation, recruitment, writing – review and editing. **Simon Laws:** conceptualisation, writing – review and editing. **Travis Cruickshank:** conceptualisation, recruitment, writing – original draft, review and editing.

## Conflicts of Interest

The authors declare no conflicts of interest.

## Supporting information

Supplementary file 1. Literature review.

Supplementary file 2.

Supplementary file 3.

## Data Availability

The data that support the findings of this study are available from the corresponding author upon reasonable request.
