# Supplementary material for: Co‐Design of a Weekly Meal Box for Neurological Conditions: Findings From Consumer and Healthcare Provider Collaborative Workshops
Source: Health Expect. 2025 Aug 28;28(5):e70412. doi: 10.1111/hex.70412 (PMC12392134; doi:10.1111/hex.70412)
Supplement: Supplementary file 1 — Literature review. [file HEX-28-e70412-s002.docx]

Literature review

To inform the work and build on from existing learnings, a comprehensive literature search was undertaken using MEDLINE, PubMed and Google Scholar across the past five years to identify existing research on meal box interventions for individuals with neurological conditions. When no research was found, the search was expanded to include all available time periods and widened to include any population. Terms included: "Meal box solution" AND/OR “nutrition" "Meal delivery service" AND/OR “nutritional intake" "Co-designed meal box “AND/OR "health outcomes" "Meal kits" AND/OR "dietary adherence" "Nutrition intervention" AND/OR "meal box" "Meal box programme" AND/OR "chronic disease management" "Ready-to-cook meals" AND/OR "health intervention" "Dietary intervention" AND/OR "meal kits" "Meal box service" AND/OR "weight management" "Food insecurity" AND/OR "meal boxes" "Meal box" AND/OR "neurological conditions" "Meal kits" AND/OR "community health intervention" "Customised meal boxes" AND/OR "patient compliance" "Nutritional needs" AND/OR "meal delivery programmes" "Home meal delivery" AND/OR "dietary improvement". The expanded literature search revealed no existing research on meal boxes and/or diet or nutrition that specifically targeted the neurological population. Although the research team were able to identify some studies undertaken in diabetes AND/OR healthy populations, none were found for neurological conditions.
