# Supplementary material for: Co‐Design of a Weekly Meal Box for Neurological Conditions: Findings From Consumer and Healthcare Provider Collaborative Workshops
Source: Health Expect. 2025 Aug 28;28(5):e70412. doi: 10.1111/hex.70412 (PMC12392134; doi:10.1111/hex.70412)

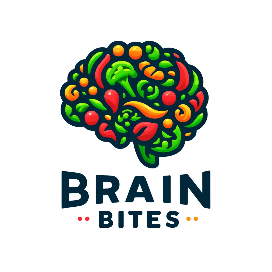
 **SIMULATION DAY INFORMATION**

Step 1 – Box receival 1^st^ photo – box before it has been unpacked

| 1 | Box weight, how easy is it to carry? | 1 | 2 | 3 | 4 | 5 | 6 | 7 | 8 | 9 | 10 |
| --- | --- | --- | --- | --- | --- | --- | --- | --- | --- | --- | --- |
| 2 | Unpacking – how easy is it to identify each meal? | 1 | 2 | 3 | 4 | 5 | 6 | 7 | 8 | 9 | 10 |
| 3 | Putting away, how easy is it to store? | 1 | 2 | 3 | 4 | 5 | 6 | 7 | 8 | 9 | 10 |

Step 2 – Recipe preparation 2^nd^ and 3^rd^ photos - ingredients before the cook and during preparation

Start Time _________________

Finish Time ­­­­­­­­­­­­­­­­­­_________________

| 1 | How easy was it to identify all the ingredients for your recipe? | 1 | 2 | 3 | 4 | 5 | 6 | 7 | 8 | 9 | 10 |
| --- | --- | --- | --- | --- | --- | --- | --- | --- | --- | --- | --- |
| 2 | Were ingredients prepared enough or too much? | 1 | 2 | 3 | 4 | 5 | 6 | 7 | 8 | 9 | 10 |
| 3 | Did ingredients look fresh and of good quality? | 1 | 2 | 3 | 4 | 5 | 6 | 7 | 8 | 9 | 10 |
| 4 | How easy was it to unwrap/open packages? | 1 | 2 | 3 | 4 | 5 | 6 | 7 | 8 | 9 | 10 |
| 5 | How much waste (packaging) was there? | 1 | 2 | 3 | 4 | 5 | 6 | 7 | 8 | 9 | 10 |

Step 3 - Cooking 4^th^ and 5^th^ photos - utensils and cooking equipment needed for the recipe and the ‘mess’ after cooking, what needed clearing/washing up

Start Time _________________

Finish Time ­­­­­­­­­­­­­­­­­­_________________

| 1 | How easy was it to follow the recipe? | 1 | 2 | 3 | 4 | 5 | 6 | 7 | 8 | 9 | 10 |
| --- | --- | --- | --- | --- | --- | --- | --- | --- | --- | --- | --- |
| 2 | How happy were you with the time it took to cook? | 1 | 2 | 3 | 4 | 5 | 6 | 7 | 8 | 9 | 10 |
| 3 | How much mess was there after cooking? | 1 | 2 | 3 | 4 | 5 | 6 | 7 | 8 | 9 | 10 |
| 4 | How physically demanding did you find it to cook your meal? |  |  |  |  |  |  |  |  |  |  |
| 5 | How mentally demanding did you find it to cook your meal? |  |  |  |  |  |  |  |  |  |  |

4. Did you make any mistakes? If yes was it easy to overcome them? Did it spoil the end result?

5. Would an assistive device make it easier to do this recipe in the future, if yes, for which step(s)?

6. How many different bits of equipment did you need/use?

7. How long did it take to cook?

Step 4 – Eating 6^th^ and 7^th^ photos - the cooked meal before eating and how much plate waste there was after eating

| 1 | How did the end result look? | 1 | 2 | 3 | 4 | 5 | 6 | 7 | 8 | 9 | 10 |
| --- | --- | --- | --- | --- | --- | --- | --- | --- | --- | --- | --- |
| 2 | Did it taste nice? [Texture, aroma, taste, appearance] | 1 | 2 | 3 | 4 | 5 | 6 | 7 | 8 | 9 | 10 |
| 3 | Was there enough or too much? | 1 | 2 | 3 | 4 | 5 | 6 | 7 | 8 | 9 | 10 |
| 4 | Are you excited to try other meals? | 1 | 2 | 3 | 4 | 5 | 6 | 7 | 8 | 9 | 10 |
| 5 | How healthy do you think the meal was? | 1 | 2 | 3 | 4 | 5 | 6 | 7 | 8 | 9 | 10 |

4. Do you think it needed anything else to be added or left out?


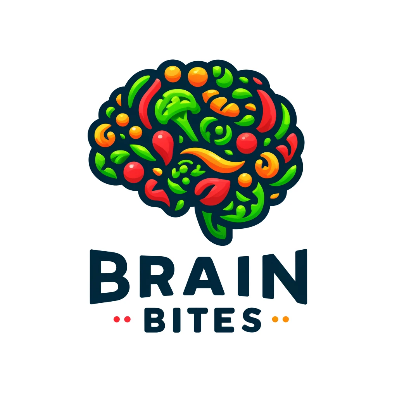

Supplement: Supplementary file 2 [file HEX-28-e70412-s001.docx]
